# Metabolic engineering of *Escherichia coli* for resveratrol production using food-grade D-xylose as a carbon source

**DOI:** 10.1093/jimb/kuag017

**Published:** 2026-07-02

**Authors:** Huy Quang Nguyen, Hoa An Thi Nguyen, Luan Luong Chu

**Affiliations:** National Key Laboratory of Enzyme and Protein Technology, Faculty of Biology, University of Science, Vietnam National University, Hanoi (VNU), 334 Nguyen Trai, Thanh Xuan, Hanoi 10000, Vietnam; National Key Laboratory of Enzyme and Protein Technology, Faculty of Biology, University of Science, Vietnam National University, Hanoi (VNU), 334 Nguyen Trai, Thanh Xuan, Hanoi 10000, Vietnam; National Key Laboratory of Enzyme and Protein Technology, Faculty of Biology, University of Science, Vietnam National University, Hanoi (VNU), 334 Nguyen Trai, Thanh Xuan, Hanoi 10000, Vietnam

**Keywords:** resveratrol, *Escherichia coli*, D-xylose, CRISPRi, metabolic engineering

## Abstract

Resveratrol is a high-value polyphenolic compound widely utilized in nutraceutical, cosmetic, and pharmaceutical applications. However, most microbial production systems rely on glucose as the primary carbon source, which limits flexibility for integrating alternative and renewable feedstocks. In this study, we developed an engineered *Escherichia coli* platform to investigate resveratrol biosynthesis under xylose-supporting conditions, using food-grade D-xylose as the carbon source. A heterologous pathway consisting of *Populus tomentosa* 4-coumarate: CoA ligase (Pt4CL) and *Arachis hypogaea* stilbene synthase (AhSTS) was introduced to convert externally supplied *p*-coumaric acid (PCA) into resveratrol. To improve precursor availability, intracellular malonyl-CoA supply was enhanced by introducing *matB* and *matC* from *Streptomyces coelicolor* A3(2) and overexpressing the acetyl-CoA carboxylase complex (ACC) from *E. coli*. Xylose assimilation was further strengthened by expressing *xylE, xylA*, and *xylB*, while carbon catabolite repression was alleviated using CRISPR interference (CRISPRi) targeting the glucose transporter gene *ptsG*. Under shake-flask conditions, the engineered strain produced up to 23.9 mg/L resveratrol from food-grade D-xylose, accompanied by near-complete xylose consumption and 93%–94% precursor conversion. This corresponded to an overall fermentation yield of approximately 12.5 mg resveratrol per g xylose consumed. Similar titers (27 mg/L) were obtained in a 5-L bioreactor, indicating stable pathway performance under controlled fermentation conditions. Overall, these results show that *E. coli* can be engineered to support efficient precursor-to-product conversion under xylose-supported conditions, providing a useful proof-of-concept framework for integrating alternative carbon sources into microbial production platforms for aromatic compounds.

**One sentence summary** An engineered *Escherichia coli* system integrates xylose utilization, malonyl-CoA pathway optimization, and CRISPRi regulation to support resveratrol biosynthesis under xylose-supported conditions.

## Graphical Abstract

**Graphical Abstract:**
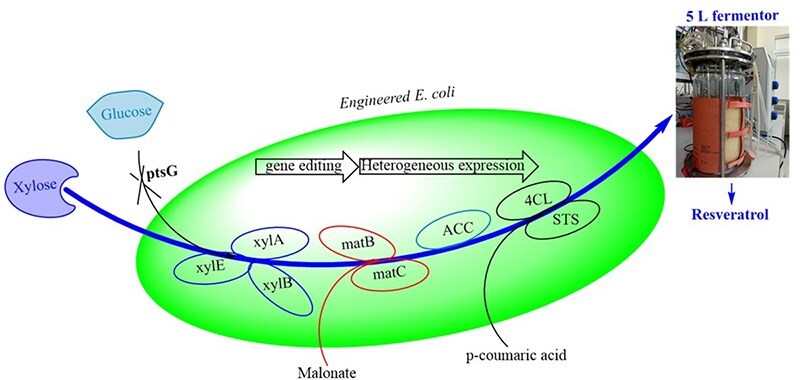

## Introduction

Resveratrol (3,4′,5-trihydroxy-trans-stilbene) is a polyphenolic compound found in over 70 plant species, including mulberries, grapes, peanuts, and cereals (Liu et al., 2025; Yu et al., 2024). It exhibits a wide range of biological activities, such as antioxidant (Huang et al., 2026), anti-inflammatory (Santos et al., 2023), anticancer (Ren et al., 2021), cardioprotective, and neuroprotective effects (Singh et al., 2013; Hung et al., 2000). Therefore, resveratrol has attracted considerable interest as a bioactive compound for pharmaceutical, nutraceutical, and functional food applications. However, the natural abundance of resveratrol in plants is generally low and highly variable, ranging from a few mg/kg to several hundred mg/kg dry weight depending on plant species, tissue type, developmental stage, and cultivation conditions. For example, the *trans*-resveratrol content in dried grape berry skin has been reported at 24.1 mg/g, whereas peanut roots contain up to 38.9 mg/kg dry weight (Chen et al., 2018; Romero et al., 2001). In mulberry fruits, resveratrol levels were determined to be approximately 50.6 mg/g, while *Polygonum cuspidatum*, one of the richest natural sources of resveratrol, contains about 295 mg/kg dry weight (Guo et al., 2026; Shrikanta et al., 2015)). Despite its occurrence in these plant sources, commercial production through plant extraction remains constrained by seasonal and geographical variability, slow biomass generation, low metabolite accumulation, and labor-intensive downstream processing. These limitations have stimulated considerable interest in developing microbial platforms as a sustainable alternative for resveratrol production (Tian and Liu 2020).

Metabolic engineering of microbial hosts has emerged as a promising strategy for sustainable and scalable resveratrol production. Various microbial hosts, including *Escherichia coli* (Kang et al., 2014; Liu et al., 2016; Park et al., 2021; Wu et al., 2017), *Corynebacterium glutamicum* (Braga et al., 2018; Kallscheuer et al., 2016), and *Saccharomyces cerevisiae* (Li et al., 2016; Shin et al., 2011), have been engineered for resveratrol production. Among these, *E. coli* offers several advantages as a production host, including rapid growth, well-characterized genetics, and a versatile metabolic engineering toolbox that encompasses plasmids, promoters, ribosome-binding site libraries, and CRISPR/Cas systems (Nonaka et al., 2025). In addition, *E. coli* supports efficient expression of plant-derived enzymes such as 4-coumarate: CoA ligase (4CL) and stilbene synthase (STS), making it a practical platform for resveratrol biosynthesis. Despite these advantages, resveratrol production in *E. coli* remains limited by insufficient precursor availability and imbalanced metabolic flux distribution. Key metabolic engineering strategies to address these limitations include modulation of regulatory genes (*tyr^R^*), attenuation of competing pathways (*trpED*), enhancement of malonyl-CoA supply through acetyl-CoA carboxylase (*ACC*) overexpression and malonate assimilation pathways (*matBC*), and reduction of carbon loss *via* disruption of acetate formation pathways (*pta-ackA*) and fatty acid biosynthesis (*fab* genes) (Lv et al., 2026; Xu et al., 2017).

Glucose is widely used as the primary carbon source for *E. coli* in industrial fermentation due to its availability and cost-effectiveness. However, increasing attention has been directed toward alternative carbon sources relevant to lignocellulosic biomass utilization and mixed-sugar bioprocessing systems (Kamasaka et al., 2025; Sohn et al., 2022). Among these, D-xylose is particularly attractive as both a major hemicellulose sugar and a commercially available food-grade sweetener approved for use in food and beverage applications. Food-grade D-xylose provides a well-defined and regulatory-compatible substrate that may facilitate translation from laboratory-scale studies to applications in food, cosmetic, and nutraceutical bioprocesses (Liang et al., 2024). Nevertheless, native *E. coli* strains utilize xylose less efficiently than glucose, as xylose uptake and catabolism are not naturally optimized for high-flux production. In mixed-sugar environments, carbon catabolite repression (CCR) further limits xylose utilization, as glucose uptake via the phosphotransferase system (PTS) suppresses the metabolism of secondary sugars (Geng et al., 2022). As a result, *E. coli* often exhibits diauxic growth, leading to prolonged fermentation time, reduced productivity, and inefficient carbon utilization. Even under xylose-only conditions, limitations in transport capacity and gene expression can restrict metabolic flux toward target compounds, highlighting the need for systematic engineering of xylose metabolism (Heo et al., 2021; Yuan et al., 2023).

**Figure 1:**
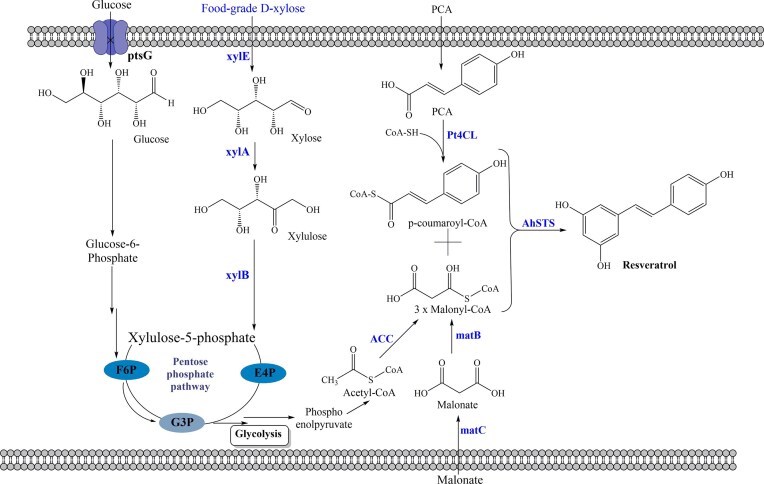
Metabolic pathway for resveratrol biosynthesis in *Escherichia coli* with enhanced malonyl-CoA supply from glucose, xylose, and malonate. The diagram illustrates the engineered genes introduced to improve xylose utilization and resveratrol biosynthesis. Enzyme abbreviations are as follows: ACC, acetyl-CoA carboxylase; matB, malonyl-CoA synthetase; matC, a putative dicarboxylate carrier protein; STS, stilbene synthase; ptsG, the glucose phosphotransferase system; xylA, xylose isomerase; xylB, xylulose kinase; and xylE, D-xylose: H(+) symporter.

To address these challenges, we focused on developing a xylose-supported production system in *E. coli* that enables systematic evaluation of carbon utilization and pathway performance. In this study, we constructed an engineered *E. coli* platform to investigate resveratrol production under xylose-supported conditions using a precursor-fed strategy (Figure 1). In this system, PCA was externally supplied as the direct aromatic precursor and converted to resveratrol *via* a two-step heterologous pathway consisting of Pt4CL and AhSTS. Because resveratrol biosynthesis requires malonyl-CoA as an extender unit, intracellular malonyl-CoA availability was enhanced by introducing the matBC pathway and overexpressing ACC. To further improve carbon utilization under xylose-based conditions, xylose assimilation was strengthened through overexpression of *xylA, xylB*, and *xylE*, while glucose-associated CCR was alleviated *via* CRISPR interference targeting *ptsG*. The engineered strains were subsequently evaluated in both shake-flask and fed-batch fermentations using food-grade D-xylose as the primary carbon source.

## Materials and methods

### Chemicals, oligonucleotides, and Reagents

Chemicals, including PCA, disodium malonate, and resveratrol standard, were purchased from Sigma-Aldrich (Vietnam). D-Xylose (100 %) was sourced from Samchun Chemicals (Seoul, Korea), and the food additive D-Xylose (100%) was purchased from NEO Foodtech Co., Ltd, and VMC GROUP, Vietnam. Acetonitrile and water HPLC-grade were used in this study from Thermo Fisher Scientific Inc. (USA). The plasmids and strains used in this research are listed in Table 1. Primers and guide RNA target sequences are summarized in Table S1. Plasmids were isolated using PureLink^TM^ Quick plasmid Miniprep Kit purchased from Thermo Fisher Scientific Inc. (USA). BioFACT™ Gel & PCR Purification System (Republic of Korea) was used to isolate DNA fragments from agarose gels. All polymerase chain reaction (PCR) products were validated *via* DNA sequencing provided by DNA sequencing Comp. (Vietnam). Oligonucleotides and synthetic long DNA fragments were ordered from PHÙ SA Genomics (Vietnam). Taq PCR master Mix Kit was purchased from Thermo Fisher Scientific Inc. (USA). Restriction enzymes, T4 DNA ligase, shrimp alkaline phosphatase, and T4 polynucleotide kinase were obtained from New England Biolabs (Hertfordshire, UK).

**Table 1:** Strains and plasmids used in this study.

Strain/plasmids	Properties/genotype	Source/Reference
**Strains**		
*E. coli* DH5*α*	F-Φ80lacZΔM15 Δ(lacZYA-argF) U169 recA1 end^A1^ hsdR17 (rK^−^, mK^+^) phoA supE44 λ^−^ thi^-1^, gyrA96 relA1	Novagen
*E. coli* BL21(DE3) (S0)	ompT hsdT hsdS (rB^-^ mB^-^) gal (DE3)	Novagen
S1	BL21 (DE3) carrying pCDF-Pt4CL-AhSTS	This study
S2	S1 carrying piBR181-matBC	This study
S3	S1 carrying piBR181-ACC	This study
S4	S1 carrying piBR181-matBC and piBR181-ACC	This study
S5	S5 carrying pETDuet-1-xylA-xylB-xylC	This study
S6	S6 carrying CRISPRi	This study
**Plasmid vectors**		
pCDFDuet-1	Double T7 promoters, CloDF13 ori, Sm^r^	Novagen
pCDF-Pt4CL	pCDF harboring *Pt4CL*, Sm^r^	This study
pCDF-Pt4CL-AhSTS	pCDF harboring *Pt4CL* and *AhSTS*, Sm^r^	This study
piBR181-matBC	piBR181 harboring *matB* and *matC* from *Streptomyces coelicolor* A3(2), Km^r^	(Ren et al., 2021)
piBR181-ACC	piBR181 harboring *ACC* from *E. coli*, Km^r^	
pETDuet-1	Double T7 promoters, f1 and pBR322 ori, Am^r^	Novagen
pETDuet-1-xylA	pETDuet-1 harboring *xylA* from *E. coli*	This study
pETDuet-1-xylA-xylB	pETDuet-1 harboring *xylA* and *xylB* from *E. coli*	This study
pETDuet-1-xylA-xylB-xylC	pETDuet-1 harboring *xylA, xylB* and *xylE from E. coli*	This study
pCRISPathBrick	pACYC184 (Cm^r^), p15A ori, *S. pyogenes* dCas9 (D10A, H840A), tracrRNA, non-targeting CRISPR spacer with *Bsa*I site	(Cress et al., 2017)
CRISPRi	pCRISPathBrick, spacer targeting *ptsG* near promoter	This study

### Media composition and culture conditions

*Escherichia coli* was grown in Lysogeny Broth (LB) medium (5 g/L yeast extract, 10 g/L tryptone, 5 g/L NaCl, pH 7.0) (Bio Basic Inc., Canada) supplemented with antibiotics at 37 °C. The antibiotics were used at approximately the following concentrations: ampicillin (100 μg/ml), kanamycin (100 μg/ml), streptomycin (50 μg/ml), and chloramphenicol (34 μg/ml) (Bio Basic Inc., Canada). *Escherichia coli* strains transformed with a plasmid including antibiotic markers were propagated on an LB agar plate [LB medium containing 2% (w/v) agar] at 37 °C. M9 minimal medium containing yeast extract (M9Y) was used for both flask and bioreactor fermentations. The defined M9Y containing 17.1 g/L Na₂HPO₄.12H₂O, 3 g/L KH₂PO₄, 0.5 g/L NaCl, 1 g/L NH₄Cl, 12 mg/L MgSO₄, 11 mg/L CaCl₂, 2 g/L glucose, and 1 g/L yeast extract.

### Plasmid and strain construction

*Escherichia coli* DH5α (Invitrogen, USA) was used for plasmid construction and propagation. *Escherichia coli* BL21 (DE3) (Invitrogen, USA) was used as an expression host for resveratrol production. To construct the expression system of resveratrol production, the genes encoding Pt4CL (GenBank: AY043495.1) and AhSTS (GenBank: AB027606.1) were codon-optimized and synthesized by Gene Universal Inc. (USA). *Pt*4CL was cloned into *Eco*RI*/Sal*I of pCDFDuet-1 vector to construct plasmid pCDFDuet-1-Pt4Cl. The codon-optimized gene AhSTS was amplified from pCDFDuet-1-AhSTS using primers AhSTS-Fw/Rv and then subcloned into *Nde*I*/Bgl*II of plasmids pCDFDuet-1-Pt4Cl to generate the plasmid pCDFDuet-1-Pt4Cl-AhSTS. The plasmid pCDFDuet-1-Pt4CL-AhSTS was introduced into *E. coli* BL21(DE3) by chemical transformation to generate the recombinant strain S1 (Table 1).

To enhance resveratrol biosynthesis by increasing intracellular malonyl-CoA availability, genes involved in malonyl-CoA formation were overexpressed. The malonate assimilation genes *matB* (GenBank: AL939112.1) and *matC* (GenBank: AL939112.1) from *Streptomyces coelicolor* A3(2), together with *ACC* genes from *E. coli* BL21(DE3). This gene cluster, which includes alpha-carboxyltransferase (*accA*, GenBank: AM946981.2), biotin carboxyl carrier protein (*accB*, GenBank: CAQ33581.1), biotin carboxylase subunit (*accC*, GenBank: AM946981.2), and beta-carboxyltransferase (*accD*, GenBank: AM946981.2), was amplified and cloned into the pIBR-181 vector as previously described (Shrestha et al., 2018). Subsequently, pIBR181-matBC and pIBR181-ACC were individually transformed into strain S1, generating strains S2 and S3, respectively. In addition, strain S4 was constructed by co-transforming both pIBR181-matBC and pIBR181-ACC into strain S1 (Table 1).

To construct the pathway for xylose consumption, the PureLinkTM Genomic DNA mini-Kit was used to isolate genomic DNA from *E. coli* K-12 MG1655. The genes encoding D-xylose: H(+) symporter (*xylE*, GenBank: AAC77001.1), xylose isomerase (*xylA*, GenBank: AAC76589.1), and xylulose kinase (*xylB*, GenBank: AAC76588.1) from *E. coli* K-12 MG1655 were cloned into the vector All in One and confirmed by Sanger sequencing. The gene encoding *xylE* was amplified using primers xylE-Fw/Rv and then subcloned into the *Blg*II*/Xho*I of plasmid pETDuet-1 to generate the plasmid pETDuet-1-xylE. Next, the gene encoding *xylB* was amplified using primers xylB-Fw/Rv and then subcloned into the *Sal*I*/Hind*III of plasmid pETDuet-1-xylE to generate the plasmid pETDuet-1-xylE-xylB. Finally, the gene encoding *xylA* was amplified using primers xylA-Fw/Rv and then subcloned into the *BamH*I*/EcoR*I of plasmid pETDuet-1-xylE-xylB to generate the plasmid pETDuet-1-xylE-xylB-xylA. Subsequently, the plasmid pETDuet-1-xylE-xylB-xylA was introduced into strain S4 to enhance xylose uptake and catabolism, yielding the recombinant strain S5 (Table 1).

### CRISPRi-mediated gene interference

Plasmids used for CRISPRi/dCas9-mediated transcriptional repression were obtained from Addgene (Plasmid #65 006) (Figure S1A) and constructed as previously reported (Cress et al., 2015). The specific targeting spacer of *ptsG* from the *E. coli* K12 genomic DNA was identified in the promoter region to prevent RNA polymerase (RNAP) elongation. The primer pairs ptsG-crRNA-Fw/Rv were used to construct CRISPRi, described in Table S1. Both primers were synthesized, phosphorylated with T_4_ polynucleotide kinase, and annealed (Cress et al., 2017). The products were then ligated into a *Bsa*I-digested, dephosphorylated, gel-purified CRISPRi plasmid backbone. *Escherichia coli* DH5α was used for cloning experiments. All CRISPRi plasmid arrays possessing a synthetic specific targeting spacer were verified by colony PCR with primer pairs cPCR-Fw/Rv (Table S1) and sequencing. Plasmids were constructed with CRISPRi and then transformed into the S5 strain using a calcium chloride and heat-shock method (Asif et al., 2017), resulting in the variant S6 strain (Table 1).

### Resveratrol production

A single colony of S1 was inoculated into 5 ml of liquid LB medium supplemented with streptomycin at 50 µg/ml and incubated overnight at 37 °C with shaking at 160 rpm. The culture was refreshed by transferring 300 µl of the overnight culture into 50 ml of M9Y. The culture flasks were shaken at 37 °C until the optical density at 600 nm (OD₆₀₀) reached 0.6, then the temperature was lowered to 30 °C. Subsequently, 0.2 mM IPTG was added to the medium to induce protein expression for 6 h. The induced cultures were further supplemented with 10 mg/L PCA and incubated at 30 °C for 48 hr. In Malonyl-CoA enhancement experiments, disodium malonate was added to the culture flasks of S2, S3, and S4 strains at a final concentration of 7,4 mg/L. Strains S5 and S6 were evaluated for resveratrol biosynthesis using food-grade D-xylose (NEO Foodtech Co., Ltd. and VMC Group, Vietnam) as the primary carbon source.

### Fermentation experiments

Fed-batch fermentation of recombinant *E. coli* strain S6 was performed in a 5-L bioreactor containing an initial working volume of 200 ml M9Y supplemented with food-grade D-xylose. A single colony was first precultured in 4 ml LB medium at 37 °C and 200 rpm overnight. This seed culture (1%, v/v) was then transferred into 100 ml LB medium and grown under the same conditions until OD₆₀₀ reached approximately 1.5. The resulting secondary seed culture (20%, v/v) was used to inoculate the bioreactor, which was maintained at 37 °C. The pH was controlled at 6.8 by automatic addition of 50% (v/v) aqueous ammonia (NH₃·H₂O). The DO level was maintained above 95% at the beginning and never <20% during the experiment. When OD₆₀₀ reached ∼2.5, the temperature was reduced to 30 °C, and 0.1 mM IPTG, 20 mg/L PCA, and 7,4 mg/L disodium malonate were added (Shrestha et al., 2018). To maintain xylose at 2 g/L, a concentrated xylose solution was fed periodically.

### Quantification of D-xylose and glucose

The concentrations of D-glucose and D-xylose in the culture supernatants were determined using commercial assay kits (Megazyme, Bray, Ireland) according to the manufacturer’s protocols (https://www.megazyme.com). All measurements were performed in 96-well microplates and quantified using an ELISA Microplate Reader (BioBase, China). All assays were performed in triplicate, and results were reported as mean ± standard deviation. D-Glucose was quantified using the D-Glucose/D-Fructose Assay Kit (K-Glu, Megazyme), which is based on sequential enzymatic reactions catalyzed by hexokinase (HK) and glucose-6-phosphate dehydrogenase (G6PDH). HK first phosphorylates glucose in the presence of ATP to form glucose-6-phosphate, which G6PDH subsequently oxidizes in the presence of NADP⁺ to form 6-phosphogluconate and NADPH. The amount of NADPH produced is stoichiometric to the glucose content and was measured by monitoring the increase in absorbance at 340 nm. Sample dilutions were adjusted to fall within the linear range of the assay, and quantification was based on a standard curve generated from glucose standards provided with the kit (Dos et al., 2016; McLoughlin et al., 2023).

D-Xylose was quantified using the D-Xylose Assay Kit (K-Xylose, Megazyme), which employs β-D-xylose dehydrogenase to oxidize D-xylose to D-xylonic acid in the presence of NAD⁺, generating NADH. Xylose mutarotase was included to ensure rapid interconversion of the α- and β-anomers. In samples containing both glucose and xylose, hexokinase and ATP were added in a pre-treatment step to remove glucose and eliminate potential interference. The reaction was incubated at 37 °C for 6 min, and the absorbance at 340 nm was measured. Xylose concentrations were calculated based on a standard calibration curve (Dos et al., 2016).

### Analytical methods

A volume of 1 ml of culture broth was mixed with an equal volume of ethyl acetate at an extract/ethyl acetate ratio of 1:1. The mixture was agitated for 12 hr at room temperature and then allowed to stand until it separated into two distinct layers. The upper ethyl acetate phase was collected into a 2-ml Eppendorf tube and evaporated to dryness. The resulting residue was dissolved in 1 ml of methanol before analysis. Resveratrol analysis was performed on an Alliance™ e2695 high-performance liquid chromatography (HPLC) system equipped with a photodiode array (PDA) detector, using a C_18_ column (250 × 4.6 mm, 5 μm particle size) at a flow rate of 0.75 ml/min. UV detection was monitored at 308 and 320 nm. The injection volume was 10 μl. The mobile phase consisted of solvent A (water containing 0.05% TFA) and solvent B (100% acetonitrile), maintained at 30 °C. An 18-min binary gradient was applied as follows: 25% B (0–1 min), 30% B (1–2.6 min), 35% B (2.6–5.2 min), 40% B (5.2–7 min), 100% B (7–11 min), held at 100% B (11–13 min), 60% B (13–14 min), 30% B (14–15 min), and 25% B (15–18 min).

### Statistical analysis

All experiments were performed using three independent biological replicates. Data are presented as mean ± standard deviation (SD). Statistical significance was evaluated using one-way analysis of variance (ANOVA) followed by Tukey’s multiple comparison test. Differences were considered statistically significant at *p* < 0.05.

## Results and discussions

### Construction of the biosynthesis pathway for resveratrol production

A heterologous resveratrol biosynthesis pathway was first established in *E. coli* BL21(DE3) by co-expressing Pt4CL and AhSTS, generating strain S1. In this precursor-fed system, PCA was externally supplied as the direct aromatic substrate for resveratrol biosynthesis at 10, 15, 20, and 25 mg/L. HPLC-PDA analysis confirmed the formation of resveratrol, with a characteristic peak at a retention time (*t_R_*) of approximately 9.6 min and a UV absorption maximum (λmax) near 309 nm, consistent with the authentic standard, while PCA was detected at *t_R_*∼6.9 min with λmax ∼305 nm (Figures S2 and S3). These results verified the functional expression of the heterologous Pt4CL-AhSTS pathway in *E. coli*.

**Figure 2:**
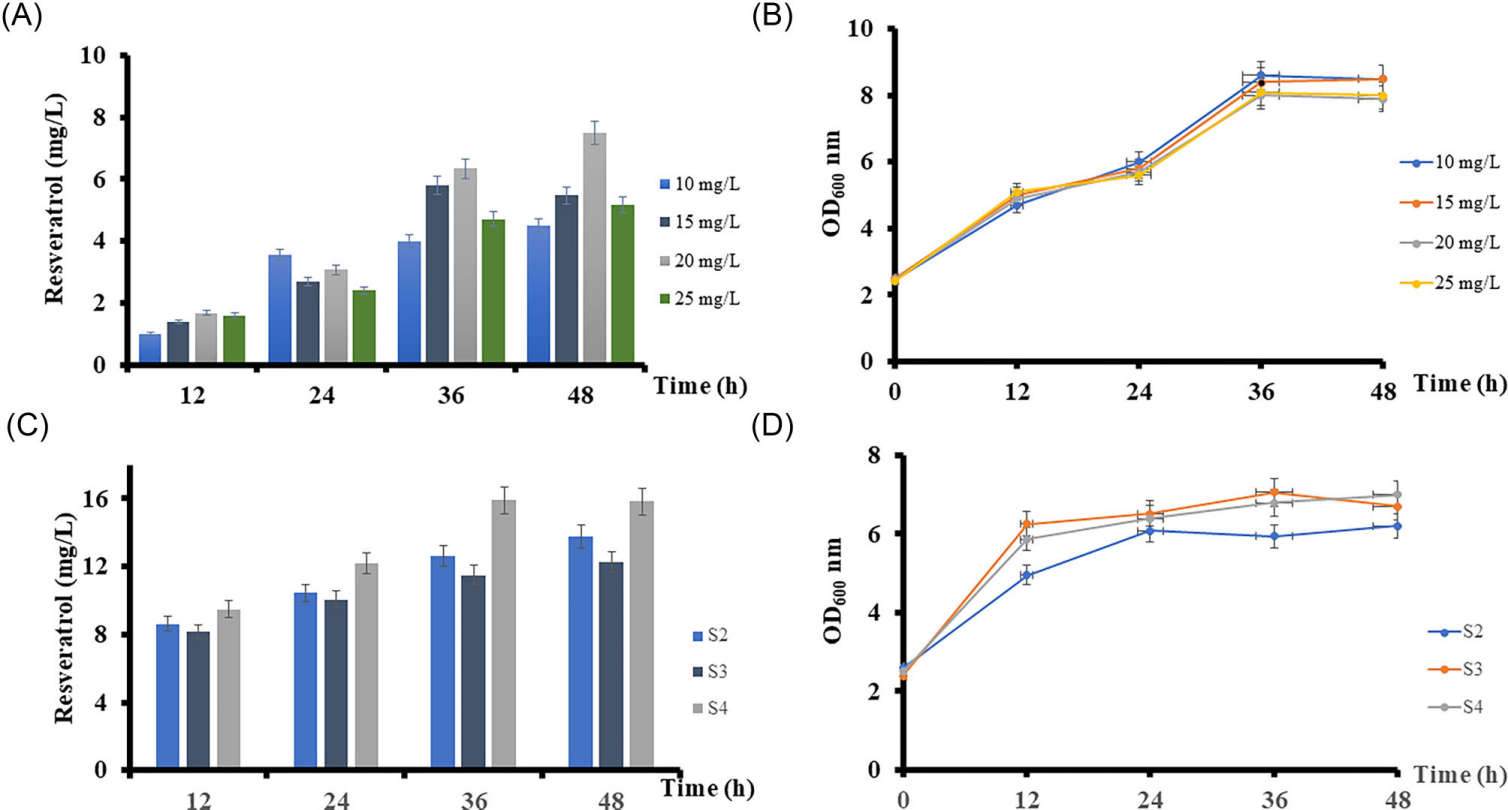
Time-course analysis of cell growth and resveratrol production in engineered *E. coli* strains. (A) Resveratrol production by strain S1 under a range of PCA concentrations (10–25 mg/L). (B) Growth profile of strain S1 at different PCA concentrations (10–25 mg/L). (C) Resveratrol titers of engineered strains S2, S3, and S4 cultured with 20 mg/L PCA. (D) Cell growth of strains S2, S3, and S4 in the presence of 20 mg/L PCA. Data represent the mean ± standard deviation (SD) of three independent biological replicates.

Resveratrol production increased with PCA concentration up to an optimal level of 20 mg/L, at which the highest titer of 7.5 mg/L was achieved after 48 h (OD₆₀₀ ∼7.9) (Figure 2A, B). Lower PCA concentrations resulted in proportionally lower titers, whereas further increasing PCA to 25 mg/L led to reduced production, suggesting possible substrate-associated inhibition or metabolic imbalance at elevated precursor levels. Residual substrate analysis showed that PCA consumption exceeded 90% across all conditions by 48 hr (Figure S4), indicating rapid precursor uptake. However, the overall molar conversion remained relatively low (∼27% based on supplemented PCA), suggesting that resveratrol accumulation is not solely limited by precursor availability. Instead, downstream metabolic constraints, particularly insufficient malonyl-CoA supply and competition from endogenous pathways, likely restrict product formation. Compared with previously reported glucose-based *E. coli* systems employing the same heterologous 4CL-STS conversion pathway, the maximum titer obtained here (7.5 mg/L) was lower than those reported by Watts et al. (105 mg/L) and Lim et al. (1.3 g/L) (Lim et al., 2011; Watts et al., 2006). In both studies, 4CL and STS enzymes were used to convert externally supplied p-coumaric acid into resveratrol, similar to the pathway implemented in the present work. However, these studies were conducted under glucose-based cultivation conditions and utilized substantially higher precursor concentrations (164.2 and 2462.4 mg/L PCA, respectively). In contrast, the present study employed only 20 mg/L PCA and focused on evaluating pathway performance in an engineered xylose-utilizing platform.

The relatively low PCA concentration was intentionally selected because the primary objective of this work was to evaluate the effectiveness of xylose-supported metabolism and carbon flux redistribution rather than to maximize resveratrol production through precursor overfeeding. In addition, high concentrations of aromatic acids such as PCA may impose a metabolic burden and inhibit cell growth, thereby complicating the assessment of pathway engineering strategies. The selected PCA range (10–25 mg/L), therefore, enabled a clearer evaluation of the contributions of enhanced xylose utilization, malonyl-CoA engineering, and CRISPRi-mediated repression of *ptsG* under conditions relevant to food-grade bioprocess development. Under this low precursor input, the observed production corresponded to approximately 27% molar conversion, confirming functional pathway activity and efficient precursor utilization. These findings indicate that improving resveratrol production requires not only precursor supplementation but also system-level metabolic optimization.

### Enhancement of resveratrol production *via* increasing the malonyl-CoA pool

Malonyl-CoA limitation is a well-recognized bottleneck in microbial resveratrol production and is not specific to xylose-supported metabolism. In native *E. coli*, exogenously supplied malonate cannot be efficiently utilized because the host lacks both an effective malonate transport system and a dedicated malonyl-CoA synthetase. Previous studies addressed this limitation by introducing the *Streptomyces coelicolor* matBC pathway, in which MatC functions as a malonate transporter and MatB catalyzes ATP-dependent conversion of malonate to malonyl-CoA (Lim et al., 2011). Additional strategies reported in the literature include overexpression of acetyl-CoA carboxylase (ACC), attenuation of fatty acid biosynthesis, and dynamic regulation of acetyl-CoA metabolism to increase intracellular malonyl-CoA availability. Therefore, the malonate utilization challenge is a general constraint in *E. coli*-based resveratrol biosynthesis rather than a limitation specific to xylose metabolism. To address the limitation of malonyl-CoA availability in *E. coli* cytosol, three engineering strategies were evaluated in the S1 background: introduction of the malonate assimilation pathway (*matBC*, strain S2), overexpression of acetyl-CoA carboxylase (ACC, strain S3), and their combination (strain S4) (Klass et al., 2025; Milke and Marienhagen 2020).

Resveratrol production followed the order S4 > S2 > S3 across all time points (Figure 2C). Strain S4 achieved the highest titer of 15.9 mg/L at 36 hr, which remained stable at 15.8 mg/L at 48 hr, representing a substantial improvement compared to the parental strain S1 (7.5 mg/L). In contrast, S2 and S3 showed moderate increases, reaching 13.8 and 12.3 mg/L, respectively. Interestingly, biomass accumulation did not correlate with product formation. Although S3 exhibited the highest OD₆₀₀, S4 produced significantly more resveratrol, indicating that metabolic flux distribution rather than cell growth determines production efficiency. This conclusion is further supported by biomass-normalized productivity, with S4 showing the highest yield per unit biomass (Figure 2D). PCA consumption profiles were similar across strains (>90% at 48 hr), suggesting that differences in resveratrol production arise from downstream flux partitioning rather than precursor uptake. The superior performance of S4 highlights the synergistic effect of combining malonate assimilation with enhanced acetyl-CoA carboxylation, leading to improved malonyl-CoA availability. However, the plateau observed after 36 hr suggests that further improvements require dynamic control of metabolic flux and reduction of competing pathways such as fatty acid biosynthesis.

### Biosynthesis of resveratrol using a recombinant xylose-consuming *E. coli* strain

**Figure 3:**
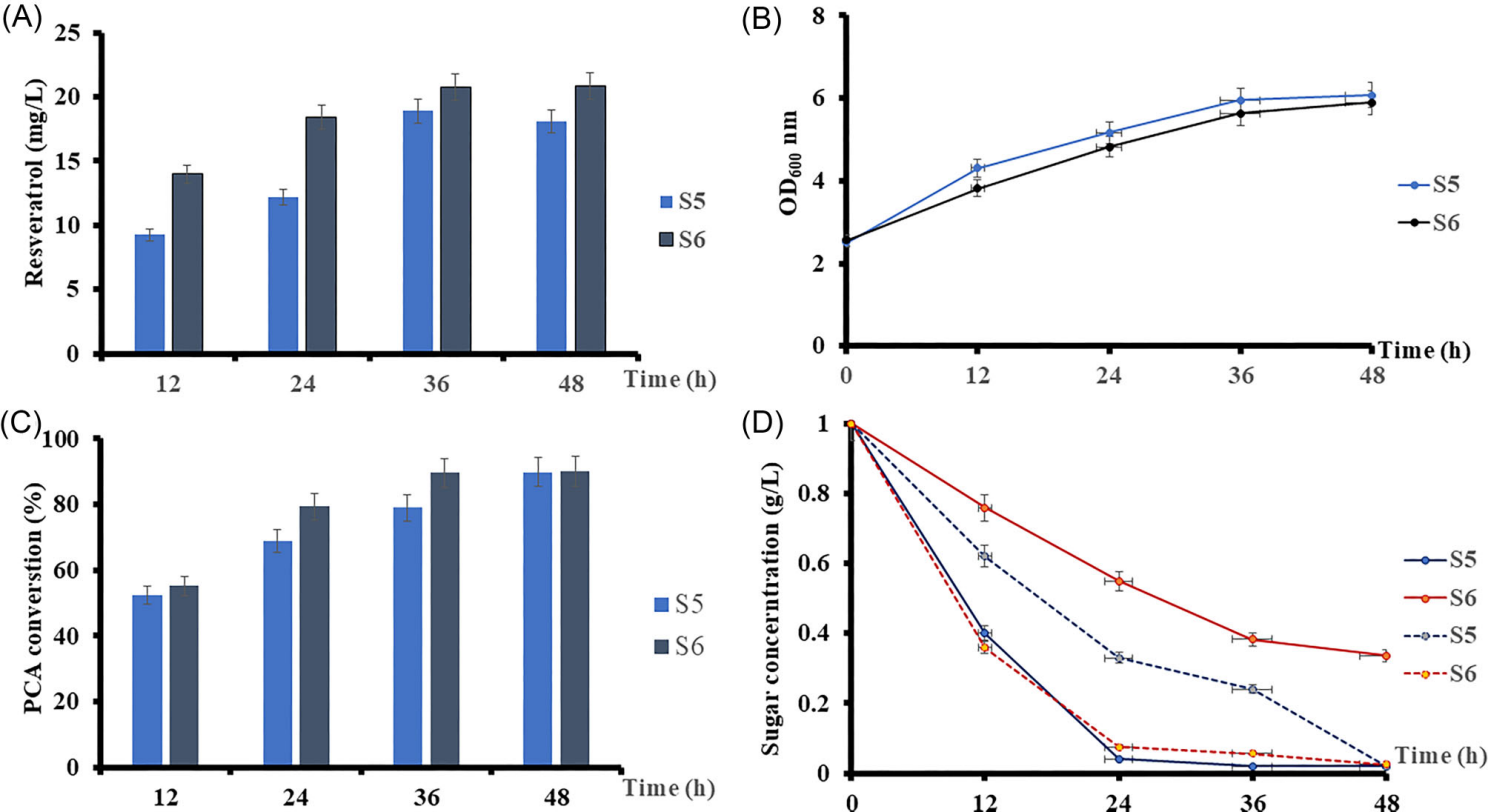
Time-course analysis of cell growth, resveratrol production, sugar consumption, and PCA conversion in strains S5 and S6. (A) Resveratrol titers during cultivation. (B) Growth curves in the presence of 20 mg/L PCA. (C) PCA conversion at different time points. (D) Residual glucose (solid lines) and xylose (dashed lines) profiles. Data represent the mean ± SD of three independent biological replicates.

To enable resveratrol production under xylose-supported conditions, strain S5 was constructed by introducing the *xylA-xylB-xylE* operon into S4. Under mixed-sugar conditions, S5 produced up to 18.9 mg/L resveratrol at 36 hr, exceeding the production level of S4 under glucose-based conditions (Figure 3A). To further improve carbon utilization, CRISPRi-mediated repression of the glucose transporter gene *ptsG* was implemented, generating strain S6. The 2% agarose gel electrophoresis results confirmed the successful construction of the CRISPathBrick-containing vector. Colony PCR of positive transformants yielded a specific 151 bp band corresponding to the CRISPRi cassette, while amplification of the synthetic gRNA region produced the expected 85 bp fragment. The presence of DNA bands at the predicted sizes, without major nonspecific products, indicated that the gRNA sequence was correctly inserted into the CRISPathBrick backbone (Figure S1B). Compared to S5, S6 exhibited consistently higher resveratrol titers at all time points, reaching 20.8 mg/L at 48 hr (Figure 3A), despite slightly lower biomass during early growth phases (Figure 3B). Despite comparable PCA conversion (∼90%) at the end of fermentation (36–48 hr) (Figure 3C), S6 achieved consistently higher resveratrol titers, suggesting more efficient carbon flux partitioning toward the target product. Moreover, sugar consumption profiles confirmed a reduced glucose preference in S6. Unlike S5, S6 exhibited higher glucose residuals (0.55–0.33 g/L at 24–48 hr) alongside accelerated xylose depletion (0.08–0.02 g/L) (Figure 3D). This shift in carbon utilization was associated with improved pathway productivity, suggesting more efficient carbon flux redistribution toward resveratrol biosynthesis. These results demonstrate that substrate utilization engineering, particularly CCR mitigation, plays a critical role in optimizing production performance in mixed-sugar systems.

### Effect of glucose-to-xylose ratio on resveratrol production

**Figure 4:**
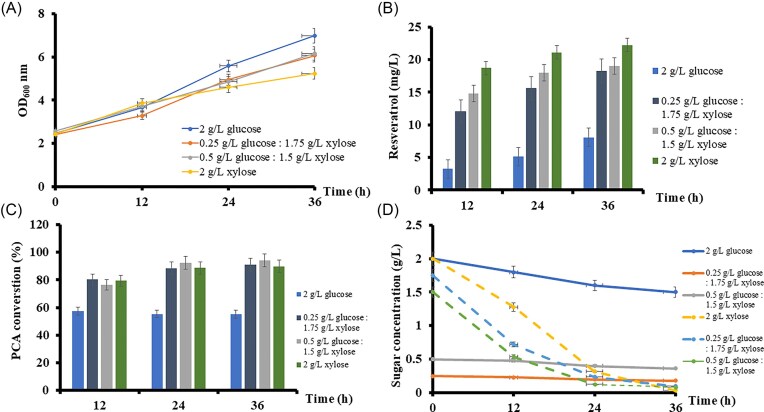
Growth, resveratrol biosynthesis, sugar consumption, and PCA conversion under different glucose/xylose feeding conditions. (A) OD_600_ profiles of strain S6 grown with 2 g/L glucose, 2 g/L xylose, or mixed glucose/xylose at 0.25:1.75 and 0.5:1.5 g/L. (B) Resveratrol titers. (C) PCA conversion. (D) Residual sugar concentrations during cultivation: residual glucose (solid lines) and xylose (dashed lines) profiles. Cultures were supplemented with 20 mg/L PCA and analyzed at 12, 24, and 36 hr. Data represent the mean ± SD of three independent biological replicates.

To further investigate the influence of carbon source composition on resveratrol biosynthesis, strain S6 was cultivated under different glucose-xylose ratios while maintaining identical precursor supplementation conditions. Strain S6 displayed carbon ratio-dependent growth, with xylose supporting the fastest early increase in OD_600_ during 0–12 hr, whereas glucose enabled the highest biomass accumulation by 36 hr, yielding the highest final OD_600_ among the tested sugar conditions (Figure 4A). Resveratrol production showed a strong dependence on carbon source composition. At 36 hr, the highest titer was observed under xylose-only conditions (22.2 mg/L), followed by mixed-sugar conditions (19.1 and 18.3 mg/L), while glucose-only conditions resulted in substantially lower production (8.1 mg/L) (Figure 4B). This pattern suggests that xylose-supported metabolism provides a more favorable intracellular environment for resveratrol biosynthesis compared to glucose-dominant conditions.

Notably, PCA conversion did not directly correlate with resveratrol accumulation across the tested conditions. At 36 hr, PCA conversion remained high under all xylose-containing conditions, ranging from approximately 90% to 94% (Figure 4C). Statistical analysis indicated that the differences among the xylose-only and mixed-sugar conditions were not significant (one-way ANOVA followed by Tukey’s test, *p* > 0.05). In contrast, PCA conversion under glucose-only conditions was significantly lower (approximately 55%; *p* < 0.05). Despite exhibiting similar PCA conversion efficiencies, the xylose-containing cultures produced different amounts of resveratrol, with the xylose-only condition achieving the highest titer (22.2 mg/L) (Figure 4D). These results suggest that precursor consumption and product formation were partially decoupled, indicating that carbon source composition influences the metabolic fate of PCA beyond its initial conversion.

Previous studies have demonstrated that simultaneous utilization of glucose and xylose can enhance microbial production of various chemicals, including methyl ketones, organic acids, and aromatic compounds (Kim et al., 2015; Li et al., 2016). In *E. coli*, engineering strategies targeting CCR, such as modification of *ptsG, crr*, or regulatory elements controlling pentose transport, have been widely applied to improve mixed-sugar utilization. For example, enabling co-utilization of glucose and xylose has been shown to enhance the production of methyl ketones and 4-hydroxymandelic acid from lignocellulosic feedstocks (Wang et al., 2018). In many cases, partial repression or deletion of *ptsG* reduces diauxic growth and promotes more efficient xylose uptake, thereby improving overall carbon utilization efficiency. The results obtained in this study are broadly consistent with these observations but also reveal a distinct metabolic behavior associated with resveratrol biosynthesis. Unlike several other fermentation products that benefit primarily from balanced sugar consumption, resveratrol production in the present system was favored under xylose-dominant conditions. This difference likely reflects the strong dependence of resveratrol biosynthesis on intracellular malonyl-CoA supply and redox balance, particularly NADPH availability, both of which are closely linked to pentose phosphate pathway (PPP) activity during xylose metabolism. Collectively, these findings suggest that the combination of precursor pathway enhancement, improved xylose utilization, and CCR mitigation *via* CRISPRi-mediated repression of *ptsG* can effectively improve resveratrol production in engineered *E. coli*. While the current system remains a precursor-fed platform, it provides a useful framework for exploring xylose-supported biosynthesis of aromatic compounds.

### Resveratrol production from food additive D-xylose in flask and fed-batch fermentation

**Fig. 5:**
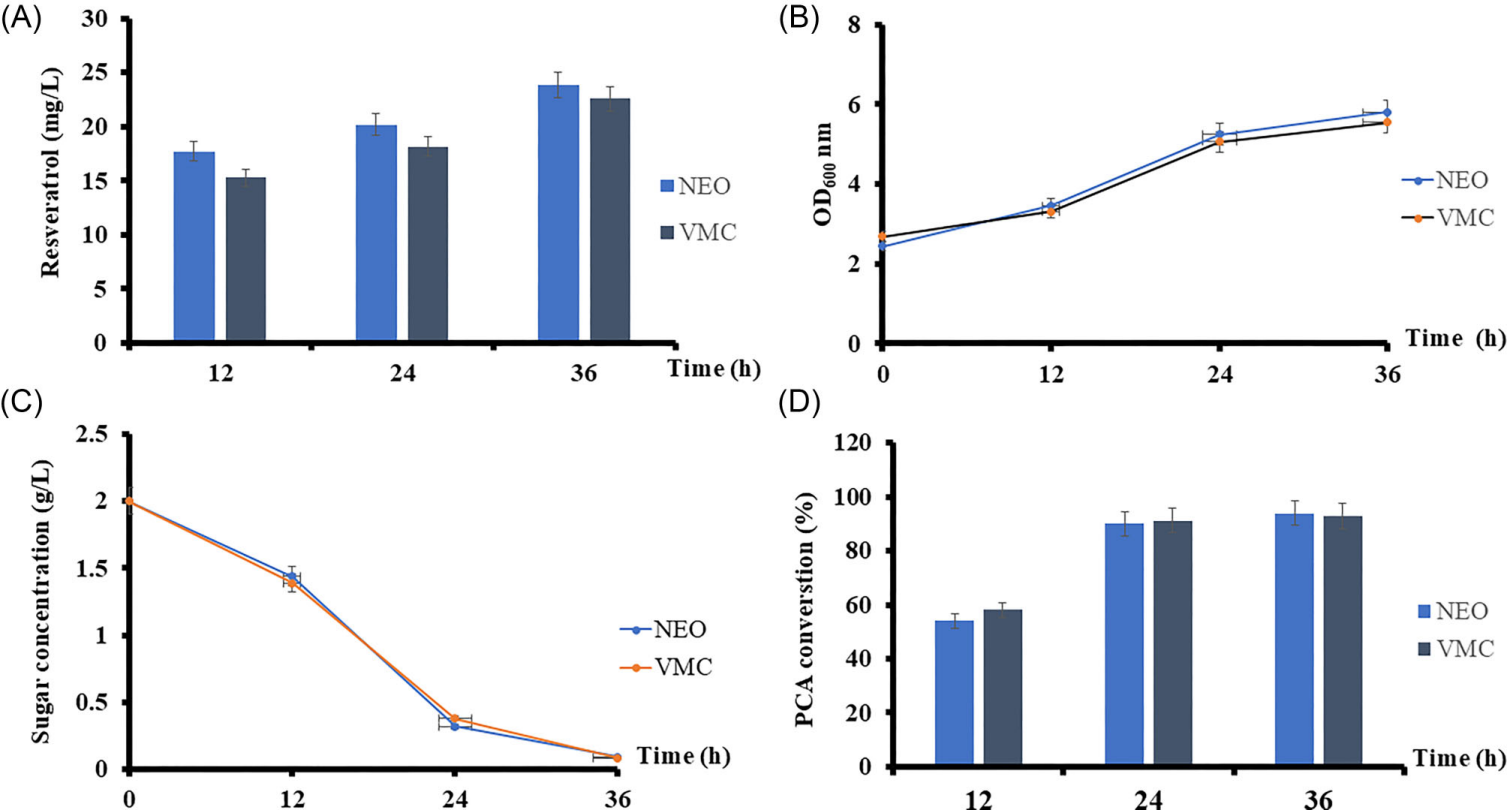
Resveratrol production from food-grade D-xylose in flask and bioreactor systems. (A) Resveratrol titers at 12, 24, and 36 hr using xylose from NEO and VMC during flask cultivation. (B) OD₆₀₀ using xylose from different suppliers (NEO and VMC). (C) Residual xylose concentrations during cultivation. (D) PCA conversion over time. Data represent the mean ± SD of three independent biological replicates.

To evaluate strain performance under xylose-supported conditions, the engineered strain S6 was cultivated in M9Y medium containing 2 g/L food-grade D-xylose obtained from two commercial suppliers (NEO and VMC). Time-course analysis showed that both xylose sources supported resveratrol biosynthesis, with NEO-derived xylose consistently yielding slightly higher titers. Resveratrol concentrations reached 17.7 and 15.3 mg/L at 12 hr, 20.2 and 18.2 mg/L at 24 hr, and 23.9 and 22.6 mg/L at 36 hr for NEO and VMC, respectively (Figure 5A). Biomass accumulation was comparable between the two conditions, with OD₆₀₀ values reaching 5.8 and 5.6 at 36 hr (Figure 5B), indicating that the observed differences in resveratrol production were not primarily attributable to growth but rather to modest variations in pathway performance. Substrate utilization profiles were highly similar in both cultures. Residual xylose decreased from 2.0 g/L to approximately 0.1 g/L at 36 hr, corresponding to near-complete substrate consumption (Figure 5C), while PCA conversion increased from 54% to 58% at 12 hr to approximately 93%–94% at 36 hr (Figure 5D). Based on the consumption of ∼1.9 g/L xylose and the production of 23.9 mg/L resveratrol, the overall yield was approximately 12.5 mg resveratrol per g xylose consumed. On a molar basis, this corresponds to ∼0.10 mmol resveratrol per mmol xylose consumed, indicating that a measurable fraction of xylose-derived carbon can be redirected toward aromatic compound biosynthesis despite competing central metabolic demands.

From a metabolic perspective, xylose assimilation *via* the *xylA-xylB-xylE* pathway channels carbon into the PPP as xylulose-5-phosphate (Molina-Vázquez et al., 2025). Increased PPP flux enhances NADPH generation and supports anabolic metabolism, thereby providing a more favorable intracellular environment for aromatic compound biosynthesis (Yuan et al., 2019). Although the STS-catalyzed condensation reaction does not directly consume NADPH, elevated PPP activity is expected to facilitate precursor formation and sustain metabolic activity during product synthesis. To assess scalability, strain S6 was further evaluated in a 5-L bioreactor using NEO-derived xylose as the sole carbon source. Under controlled conditions, the culture reached an OD₆₀₀ of 10.5 and produced 27 mg/L resveratrol at 36 h, accompanied by 98% xylose consumption and 96.9% PCA conversion. The increased biomass relative to shake-flask cultivation indicates improved growth under controlled aeration and agitation. Although the 5-L bioreactor supported substantially higher biomass and a slightly higher resveratrol titer than shake-flask cultivation, the gain in volumetric production was relatively limited compared with the increase in cell density. This observation suggests that factors beyond biomass accumulation influence resveratrol production under the current conditions. Intracellular precursor availability, particularly malonyl-CoA supply, and competing metabolic fluxes may therefore contribute to the limited improvement in titer.

Previous studies have primarily reported resveratrol production in glucose-based systems, often achieving higher titers under extensively optimized conditions (Lim et al., 2011; Watts et al., 2006). However, these systems differ substantially from the present xylose-supported, precursor-fed platform in both carbon source and process configuration. The present study demonstrates that food-grade D-xylose can support resveratrol biosynthesis with high precursor conversion efficiency under xylose-supported conditions. While the current system remains a precursor-fed platform with relatively low volumetric titers, it provides a useful framework for investigating the integration of alternative carbon sources into microbial production systems. These results indicate that the combination of enhanced xylose assimilation, precursor pathway optimization, and CRISPRi-mediated mitigation of CCR enables stable resveratrol production under xylose-supported conditions. This work establishes a foundation for further metabolic engineering toward more efficient and integrated biosynthetic systems for aromatic compounds.

## Conclusion

In this study, an engineered *E. coli* platform was developed to investigate resveratrol production under xylose-supported conditions using food-grade D-xylose as the primary carbon source. Shake-flask cultivation demonstrated efficient conversion of externally supplied PCA into resveratrol, reaching titers of approximately 24 mg/L with near-complete xylose consumption and high precursor conversion efficiency. Scale-up fermentation in a 5-L bioreactor further confirmed the stability of the system, achieving up to 27 mg/L resveratrol under controlled conditions. Metabolic analysis suggests that xylose assimilation through the PPP provides a favorable intracellular environment for aromatic compound biosynthesis by supporting precursor formation and redox balance. While the current system employs a precursor-fed strategy and achieves moderate volumetric titers, further improvements in resveratrol production are likely to benefit from optimization of intracellular metabolic flux, particularly malonyl-CoA availability and competing pathways. Overall, this study provides a proof-of-concept framework for integrating xylose metabolism into microbial production systems for aromatic compounds and highlights key directions for future metabolic engineering.

## Supplementary Material

kuag017_Supplemental_File

## References

[bib1] Asif A., Mohsin H., Tanvir R., Rehman Y. (2017). Revisiting the mechanisms involved in calcium chloride induced bacterial transformation. Frontiers in Microbiology, 8, 2169. 10.3389/fmicb.2017.02169 29163447 PMC5681917

[bib2] Braga A., Oliveira J., Silva R., Ferreira P., Rocha I., Kallscheuer N., Marienhagen J., Faria N. (2018). Impact of the cultivation strategy on resveratrol production from glucose in engineered *Corynebacterium glutamicum*. Journal of Biotechnology, 265, 70–75. 10.1016/j.jbiotec.2017.11.006 29141192

[bib3] Chen J., Jiang X. X., Yang G., Bi Y. L., Liu W. (2018). Green and efficient extraction of resveratrol from peanut roots using deep eutectic solvents. Journal of Chemistry, 2018, 1. 10.1155/2018/4091930

[bib4] Cress B. F., Leitz Q. D., Kim D. C., Amore T. D., Suzuki J. Y., Linhardt R. J., Koffas M. A. (2017). CRISPRi-mediated metabolic engineering of *E. coli* for O-methylated anthocyanin production. Microbial Cell Factories, 16(1), 10. 10.1186/s12934-016-0623-3 28095853 PMC5240198

[bib5] Cress B. F., Toparlak Ö. D., Guleria S., Lebovich M., Stieglitz J. T., Englaender J. A., Jones J. A., Linhardt R. J., Koffas M. A. (2015). CRISPathBrick: Modular combinatorial assembly of type II-A CRISPR arrays for dCas9-mediated multiplex transcriptional repression in *E. coli*. ACS Synthetic Biology, 4(9), 987–1000. 10.1021/acssynbio.5b00012 25822415

[bib6] Dos Reis T. F, de Lima P. B., Parachin N. S., Mingossi F. B., de Castro Oliveira J. V., Ries L. N., Goldman G. H. (2016). Identification and characterization of putative xylose and cellobiose transporters in *Aspergillus nidulans*. Biotechnology for Biofuels, 9, 204. 10.1186/s13068-016-0611-1 27708711 PMC5037631

[bib7] Geng B., Jia X., Peng X., Han Y. (2022). Biosynthesis of value-added bioproducts from hemicellulose of biomass through microbial metabolic engineering. Metabolic Engineering Communications, 15, e00211. 10.1016/j.mec.2022.e00211 36311477 PMC9597109

[bib8] Guo Y., Wan S., Gu Y., He T., Chen Z., Qu X., Quan J., Ma J., Hamid I. A. A. (2026). Optimization of extraction and antioxidant activities of resveratrol from *Polygonum cuspidatum* by ultrasound-assisted natural deep eutectic solvent method. Molecules, 31(3), 492. 10.3390/molecules31030492 41683469 PMC12899112

[bib9] Heo J. M., Kim H. J., Lee S. J. (2021). Efficient anaerobic consumption of D-xylose by *E. coli* BL21(DE3) via xylR adaptive mutation. BMC Microbiology, 21(1), 332. 10.1186/s12866-021-02395-9 34872501 PMC8647362

[bib10] Huang T., Chen X., Chen D., Yu B., Yan H., Zheng P., Luo J., Huang Z. (2026). Comparative study of lipophilicity, cell membrane permeability, and intracellular antioxidant capacity of resveratrol and pterostilbene. The Journal of Nutritional Biochemistry, 147, 110095. 10.1016/j.jnutbio.2025.110095 40915507

[bib11] Hung L. M., Chen J. K., Huang S. S., Lee R. S., Su M. J. (2000). Cardioprotective effect of resveratrol, a natural antioxidant derived from grapes. Cardiovascular Research, 47(3), 549–555. 10.1016/S0008-6363(00)00102-4 10963727

[bib12] Kallscheuer N., Vogt M., Stenzel A., Gätgens J., Bott M., Marienhagen J. (2016). Construction of a *Corynebacterium glutamicum* platform strain for the production of stilbenes and (2S)-flavanones. Metabolic Engineering, 38, 47–55. 10.1016/j.ymben.2016.06.003 27288926

[bib13] Kamasaka K., Marcello L., Domingues L., Hasunuma T. (2025). Harnessing glycerol for secondary metabolite biosynthesis in microorganisms. World Journal of Microbiology and Biotechnology, 41(9), 325. 10.1007/s11274-025-04537-x 40952535 PMC12436559

[bib14] Kang S. Y., Lee J. K., Choi O., Kim C. Y., Jang J. H., Hwang B. Y., Hong Y. S. (2014). Biosynthesis of methylated resveratrol analogs through the construction of an artificial biosynthetic pathway in *E. coli*. BMC Biotechnology, 14, 67. 10.1186/1472-6750-14-67 25033820 PMC4118633

[bib15] Kim S. M., Choi B. Y., Ryu Y. S., Jung S. H., Park J. M., Kim G. H., Lee S. K. (2015). Simultaneous utilization of glucose and xylose via novel mechanisms in engineered *Escherichia coli*. Metabolic Engineering, 30, 141–148. 10.1016/j.ymben.2015.05.002 26045332

[bib16] Klass S. H., Wesselkamper M., Cowan A. E., Lee N., Lanclos N., Cheong S., Wang Z., Chen Y., Gin J. W., Petzold C. J., Keasling J. D. (2025). Engineering controllable alteration of malonyl-CoA levels to enhance polyketide production. Nature Chemical Biology, 21(8), 1214–1225. 10.1038/s41589-025-01911-6 40500421 PMC12303837

[bib17] Li F. F., Zhao Y., Li B. Z., Qiao J. J., Zhao G. R. (2016). Engineering *Escherichia coli* for production of 4-hydroxymandelic acid using glucose-xylose mixture. Microbial Cell Factories, 15, 90. 10.1186/s12934-016-0489-4 27234226 PMC4884394

[bib18] Li M., Schneider K., Kristensen M., Borodina I., Nielsen J. (2016). Engineering yeast for high-level production of stilbenoid antioxidants. Scientific Reports, 6, 36827. 10.1038/srep36827 27833117 PMC5105057

[bib19] Liang Q., Zhang F., Li Y., Zhang X., Li J., Yang P., Qi Q. (2015). Comparison of individual component deletions in a glucose-specific phosphotransferase system revealed their different applications. Scientific Reports, 5, 13200. 10.1038/srep13200 26285685 PMC4541071

[bib20] Liang Z., Zheng K., Xie G., Luo X., Li H. (2024). Sugar utilization-associated food-grade selection markers in lactic acid bacteria and yeast. Polish Journal of Microbiology, 73(1), 3–10. 10.33073/pjm-2024-011 38437472 PMC10911659

[bib21] Lim C. G., Fowler Z. L., Hueller T., Schaffer S., Koffas M. A. (2011). High-yield resveratrol production in engineered *Escherichia coli*. Applied and Environmental Microbiology, 77(10), 3451–3460. 10.1128/AEM.02186-10 21441338 PMC3126431

[bib22] Liu X., Lin J., Hu H., Zhou B., Zhu B. (2016). De novo biosynthesis of resveratrol by site-specific integration of heterologous genes in *Escherichia coli*. FEMS Microbiology Letters, 363(8), fnw061. 10.1093/femsle/fnw061 26976851

[bib23] Liu X., Pei J., Li J., Zhu H., Zheng X., Zhang X., Ruan B., Chen L. (2025). Recent advances in resveratrol derivatives: Structural modifications and biological activities. Molecules, 30(4), 958. 10.3390/molecules30040958 40005268 PMC11858244

[bib24] Lv J., An J., Sun Z., Zhao G., Ding X., Deng X., Tan H., Cai J., Liang L., Liu R. (2026). Engineering *Escherichia coli* for robust co-utilization of glucose and xylose enables high-titer succinate production from lignocellulosic hydrolysates. Synthetic and Systems Biotechnology, 13, 14–24. 10.1016/j.synbio.2026.01.006 41624986 PMC12857410

[bib25] McLoughlin C., McKie V. A., McCleary B. V. (2023). Validation of the test method-determination of available carbohydrates in cereal and cereal products, dairy products, vegetables, fruit, and related food products and animal feeds: Collaborative study, final action 2020.07. Journal of AOAC International, 106(2), 370–383. 10.1093/jaoacint/qsac116 36179081 PMC9978598

[bib26] Milke L., Marienhagen J. (2020). Engineering intracellular malonyl-CoA availability in microbial hosts and its impact on polyketide and fatty acid synthesis. Applied Microbiology and Biotechnology, 104(14), 6057–6065. 10.1007/s00253-020-10643-7 32385515 PMC7316851

[bib27] Molina-Vázquez E. R., Caspeta L., Gosset G., Martínez A. (2025). Tailoring *Escherichia coli* BL21 (DE3) for preferential xylose utilization via metabolic and regulatory engineering. Applied Microbiology and Biotechnology, 109(1), 54. 10.1007/s00253-025-13430-4 40019617 PMC11870883

[bib28] Nonaka D., Kishida M., Hirata Y., Mori A., Kondo A., Mori Y., Noda S., Tanaka T. (2025). Metabolic engineering for resveratrol production based on modularization of metabolic pathways in *Escherichia coli*. Journal of Agricultural and Food Chemistry, 73(19), 11878–11888. 10.1021/acs.jafc.5c00804 40305421

[bib29] Park J. Y., Lim J. H., Ahn J. H., Kim B. G. (2021). Biosynthesis of resveratrol using metabolically engineered *Escherichia coli*. Applied Biological Chemistry, 64, 20. 10.1186/s13765-021-00595-5

[bib30] Ren B., Kwah M. X., Liu C., Ma Z., Shanmugam M. K., Ding L., Xiang X., Ho P. C., Wang L., Ong P. S., Goh B. C. (2021). Resveratrol for cancer therapy: Challenges and future perspectives. Cancer Letters, 515, 63–72. 10.1016/j.canlet.2021.05.001 34052324

[bib31] Romero P. A. I., Lamuela R. R. M., Andres L. C., de La Torre B. M. C. (2001). Method for the quantitative extraction of resveratrol and piceid isomers in grape berry skins. Effect of powdery mildew on the stilbene content. Journal of Agricultural and Food Chemistry, 49(1), 210–215. 10.1021/jf000745o 11170579

[bib32] Santos M. A., Franco F. N., Caldeira C. A., de Araújo G. R., Vieira A., Chaves M. M. (2023). Resveratrol has its antioxidant and anti-inflammatory protective mechanisms decreased in aging. Archives of Gerontology and Geriatrics, 107, 104895. 10.1016/j.archger.2022.104895 36525827

[bib33] Shin S. Y., Han N. S., Park Y. C., Kim M. D., Seo J. H. (2011). Production of resveratrol from p-coumaric acid in recombinant Saccharomyces cerevisiae expressing 4-coumarate:Coenzyme A ligase and stilbene synthase genes. Enzyme and Microbial Technology, 48(1), 48–53. 10.1016/j.enzmictec.2010.09.004 22112770

[bib34] Shrestha A., Pandey R. P., Pokhrel A. R., Dhakal D., Chu L. L., Sohng J. K. (2018). Modular pathway engineering for resveratrol and piceatannol production in engineered *Escherichia coli*. Applied Microbiology and Biotechnology, 102(22), 9691–9706. 10.1007/s00253-018-9323-8 30178203

[bib35] Shrikanta A., Kumar A., Govindaswamy V. (2015). Resveratrol content and antioxidant properties of underutilized fruits. Journal of Food Science and Technology, 52(1), 383–390. 10.1007/s13197-013-0993-z 25593373 PMC4288802

[bib36] Singh N., Agrawal M., Doré S. (2013). Neuroprotective properties and mechanisms of resveratrol in in vitro and in vivo experimental cerebral stroke models. ACS Chemical Neuroscience, 4(8), 1151–1162. 10.1021/cn400094w 23758534 PMC3750679

[bib37] Sohn Y. J., Son J., Lim H. J., Lim S. H., Park S. J. (2022). Valorization of lignocellulosic biomass for polyhydroxyalkanoate production: Status and perspectives. Bioresource Technology, 360, 127575. 10.1016/j.biortech.2022.127575 35792330

[bib38] Tian B., Liu J. (2020). Resveratrol: A review of plant sources, synthesis, stability, modification and food application. Journal of the Science of Food and Agriculture, 100(4), 1392–1404. 10.1002/jsfa.10152 31756276

[bib39] Wang X., Goh E. B., Beller H. R. (2018). Engineering *E. coli* for simultaneous glucose-xylose utilization during methyl ketone production. Microbial Cell Factories, 17(1), 12. 10.1186/s12934-018-0862-6 29374483 PMC5787283

[bib40] Watts K. T., Lee P. C., Schmidt-Dannert C. (2006). Biosynthesis of plant-specific stilbene polyketides in metabolically engineered *Escherichia coli*. BMC Biotechnology, 6, 22. 10.1186/1472-6750-6-22 16551366 PMC1435877

[bib41] Wu J., Zhou P., Zhang X., Dong M. (2017). Efficient de novo synthesis of resveratrol by metabolically engineered *Escherichia coli*. Journal of Industrial Microbiology and Biotechnology, 44(7), 1083–1095. 10.1007/s10295-017-1937-9 28324236

[bib42] Xu Q., Bai F., Chen N., Bai G. (2017). Gene modification of the acetate biosynthesis pathway in *Escherichia coli* and implementation of the cell recycling technology to increase L-tryptophan production. PLoS One, 12(6), e0179240. 10.1371/journal.pone.0179240 28622378 PMC5473561

[bib43] Yu X., Jia Y., Ren F. (2024). Multidimensional biological activities of resveratrol and its prospects and challenges in the health field. Frontiers in Nutrition, 11, 1408651. 10.3389/fnut.2024.1408651 38933889 PMC11199730

[bib44] Yuan X., Cao J., Wang R., Han Y., Zhu J., Lin J., Yang L., Wu M. (2023). Genetically engineering *Escherichia coli* to produce xylitol from corncob hydrolysate without lime detoxification. Molecules, 28(4), 1550. 10.3390/molecules28041550 36838538 PMC9967598

[bib45] Yuan X., Wang J., Lin J., Yang L., Wu M. (2019). Efficient production of xylitol by the integration of multiple copies of xylose reductase gene and the deletion of Embden–Meyerhof–Parnas pathway-associated genes to enhance NADPH regeneration in *Escherichia coli*. Journal of Industrial Microbiology and Biotechnology, 46(8), 1061–1069. 10.1007/s10295-019-02169-3 31025135

