# Supplementary material for: Metabolic engineering of Escherichia coli for resveratrol production using food-grade D-xylose as a carbon source
Source: J Ind Microbiol Biotechnol. 2026 Jul 2;53:kuag017. doi: 10.1093/jimb/kuag017 (PMC13358167; doi:10.1093/jimb/kuag017)
Supplement: kuag017_Supplemental_File [file kuag017_supplemental_file.docx]

*Original paper*

**Metabolic engineering of *Escherichia coli* for resveratrol production using food-grade D-xylose as a carbon source**

Huy Quang Nguyen^1^, Hoa An Thi Nguyen^1^, Luan Luong Chu^1,^*

*^1^ National Key Laboratory of Enzyme and Protein Technology, Faculty of Biology, University of Science, Vietnam National University, Hanoi (VNU), 334 Nguyen Trai, Thanh Xuan, Hanoi 10000, Vietnam*

*****Corresponding author: Luan Luong Chu, Ph.D.

Tel: (84) 0243-8584615/985464929; Fax: (84) 0243-8523061

Email: luancl@vnu.edu.vn

*Disclosures*: The authors declare no competing interests.

*One-Sentence Summary*: An engineered *E. coli* system integrates xylose utilization, malonyl-CoA pathway optimization, and CRISPRi regulation to support resveratrol biosynthesis under xylose-supported conditions.

**Supplementary Information**

**Table S1.** Primers used in this study

| **Name** | **Nucleotide Sequence (5’ → 3’)** | **Restriction Enzyme** |
| --- | --- | --- |
| xylE-Fw | GCATAGATCTTTACAGCGTAGCAGTTTGTTGTG | *Blg*II |
| xylE-Rv | GCTCGAGATGAATACCCAGTATAATTCCAGTTATATATTTTCG | *Xho*I |
| xylB-Fw | GCGTCGACAAGGAGAGCTAGATGTATATCGGGATAGATCTTGG | *Sal*I |
| xylB-Rv | GACAAAGCTTTTACGCCATTAATGGCAGAAG | *Hind*III |
| xylA-Fw | CTGGGATCCTTATTTGTCGAACAGATAATGGTTTACC | *BamH*I |
| xylA-Rv | CTGGAATTCATGCAAGCCTATTTTGACCAGC | *EcoR*I |
| ptsG-crRNA-Fw | AAACCGTGAGAACGTAAAAAAAGCACCCATACTCGTTTTAGAGCTATGCTGTTTTGAATGGTCCCA |  |
| ptsG-crRNA-Rv | GTTTTGGGACCATTCAAAACAGCATAGCTCTAAAAC GAGTATGGGTGCTTTTTTTACGTTCTCACG |  |
| cPCR-Fw | GACAAAAATAGTCTACGAGGTTTTAG |  |
| cPCR-Rv | GAGTCCTATGAGCTTCCGAGACAG |  |
| *Note: Underline indicates restriction enzyme* | | |

**Fig. S1.**

**
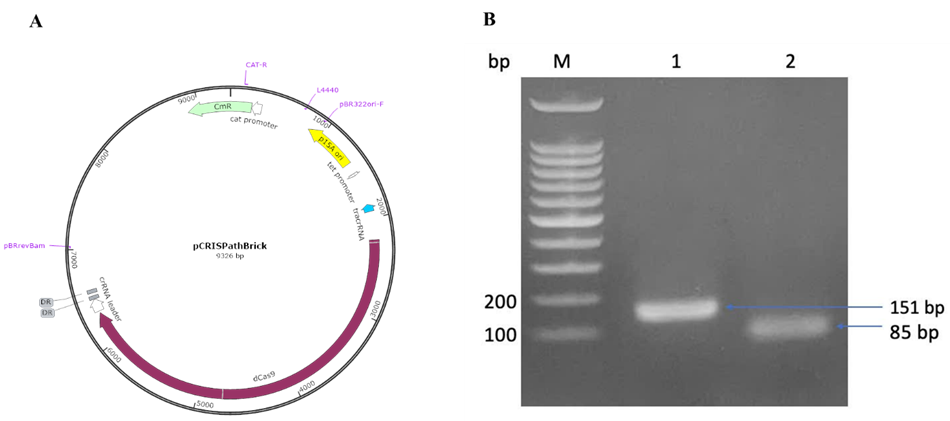
**

**Fig. S1.** **Structure of CRISPRi Plasmid used in this study and analysis of colony PCR (A)** pCRISPathBrick vector (9236 bp) for expression of *S. pyogenes* dCas9, tracrRNA, crRNA leader, and two direct repeats possessing a single *Bsa*I site for inserting user-defined spacer-repeat bricks. Cm^R^, chloramphenicol resistance gene; p15A ori, the medium-copy number p15A origin of replication; *tet* promoter, *E. coli* promoter for tetracycline efflux protein gene; *cat* promoter, promoter of *E. coli* *cat* gene encoding chloramphenicol acetyltransferase. **(B)** 2% agarose gel analysis of colony PCR. Lane M, 100 bp DNA ladder marker; Lane 1, colony PCR of CRISPRi ̣ (151 bp); Lane 2, the synthetic gRNA obtained from a positive clone of CRISPathBrick is 85 bp.

**Fig. S2.**

**
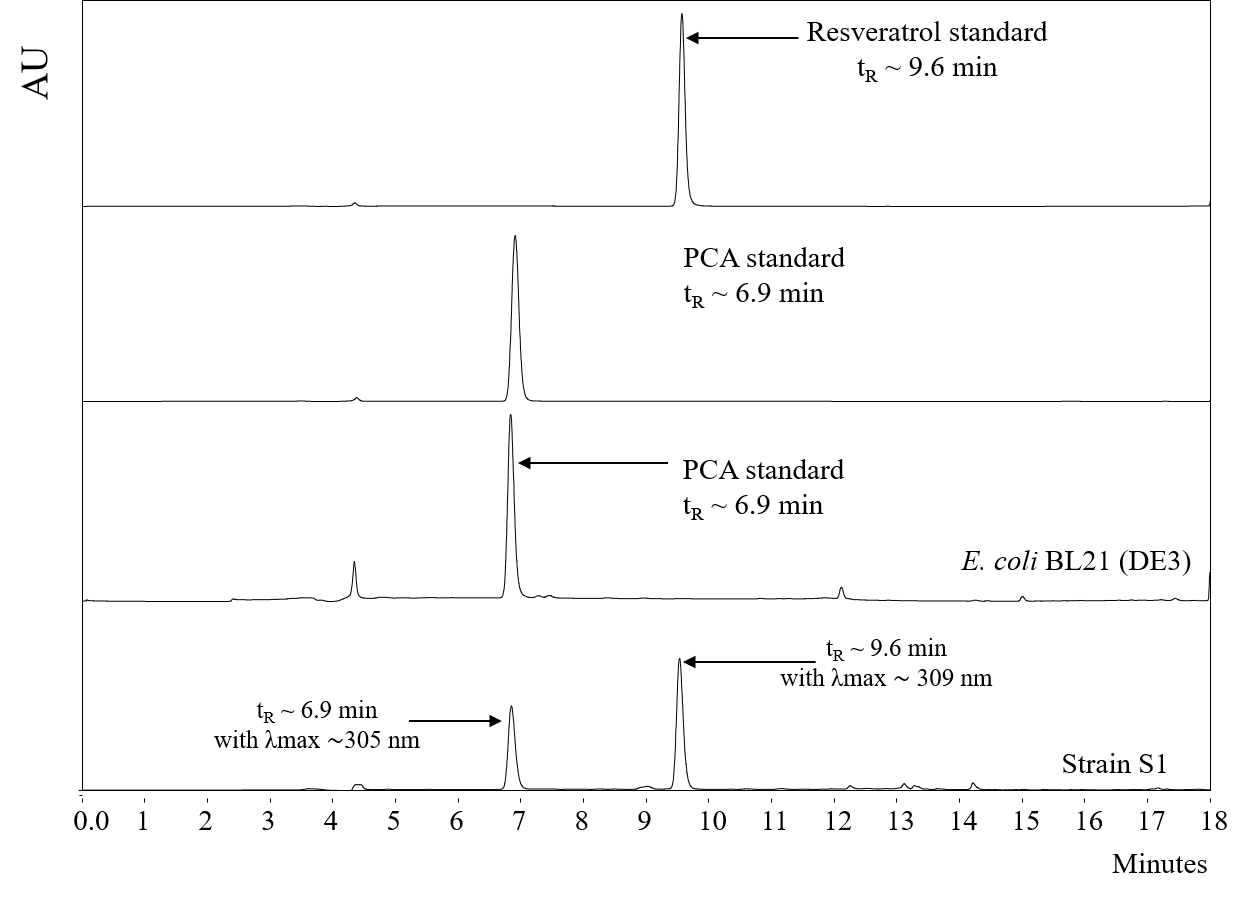
**

**Fig. S2**. **HPLC chromatograms of standards and culture extracts for resveratrol identification**. Resveratrol and p-coumaric acid (PCA) standards showed peaks at t_R_ ~ 9.6 min and t_R_ ~ 6.9 min, respectively. No corresponding resveratrol peak was detected in the *E. coli* BL21(DE3) control. In contrast, strain S1 exhibited peaks at t_R_ ~6.9 min and tR ~9.6 min.

**Fig. S3**.


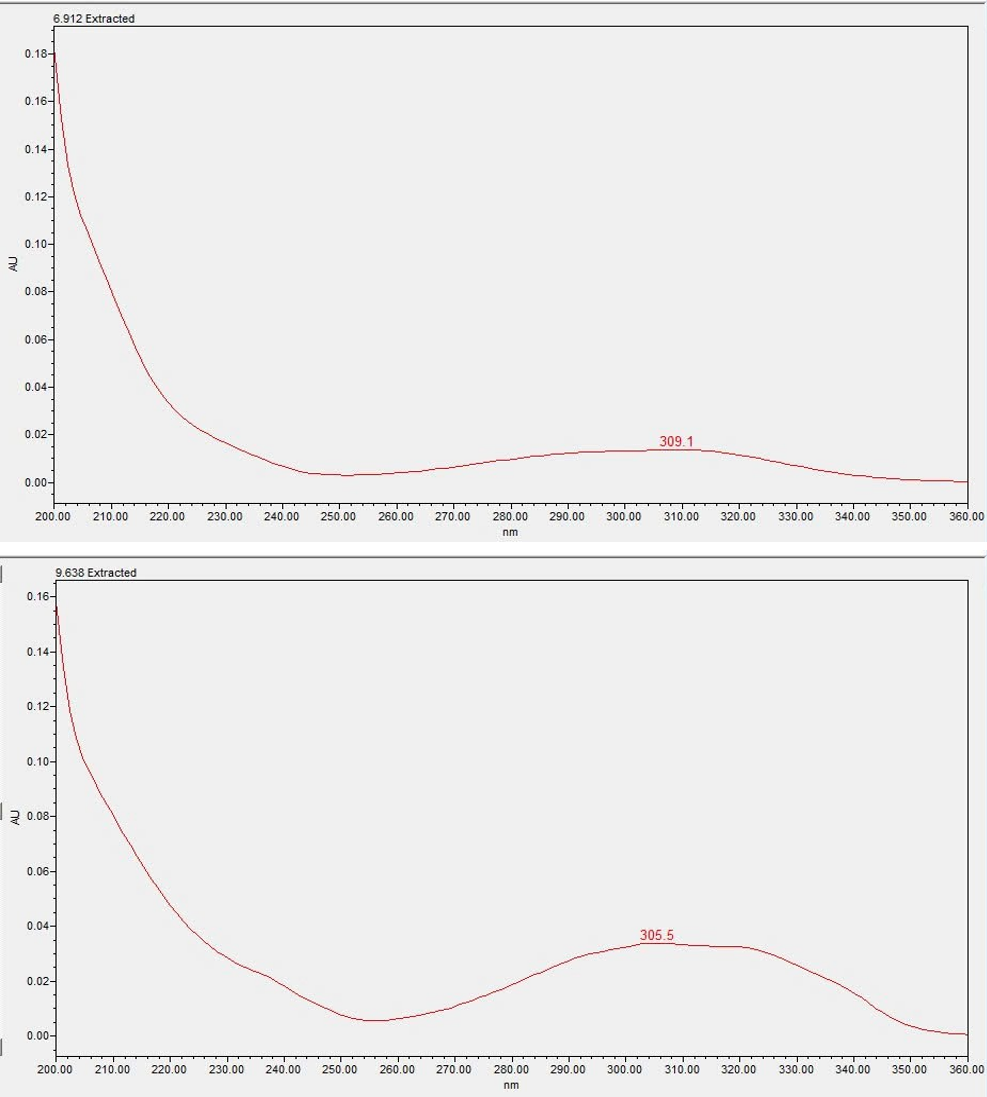


**Fig. S3**. **UV spectra of standards and culture extracts for resveratrol identification**. The extracted UV spectra showed λmax ~305 nm for the 6.9-min peak and λmax ~309 nm for the 9.6-min peak, consistent with PCA and resveratrol, respectively.

**Fig. S4.**

**
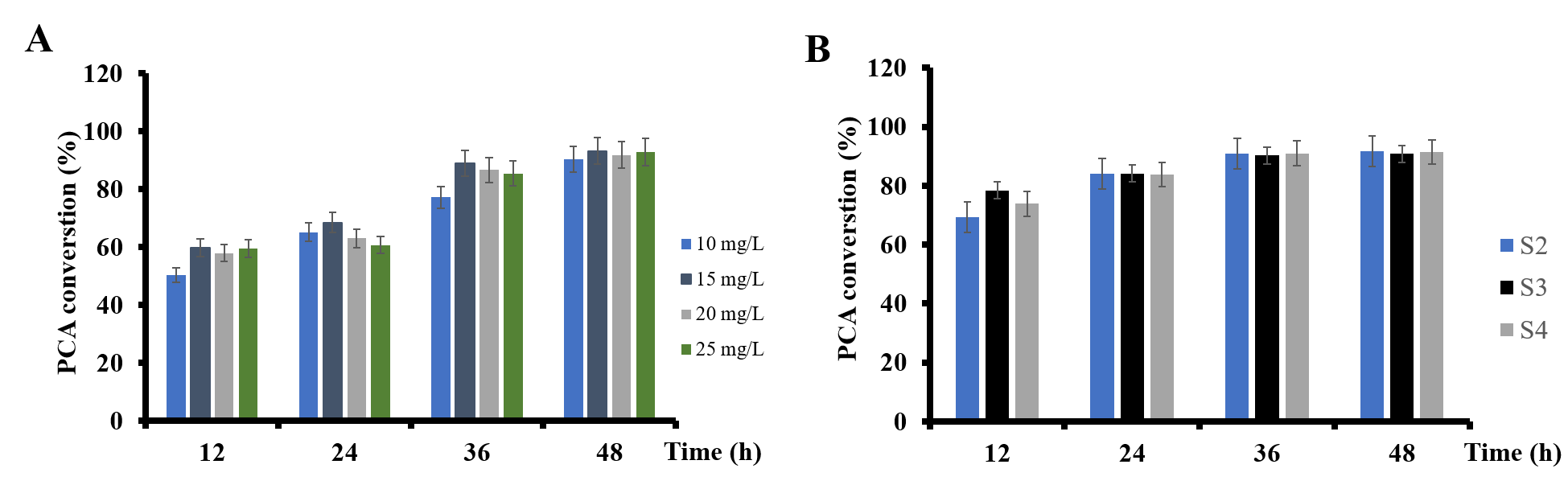
**

**Fig. S4. PCA conversion profiles of engineered strains during resveratrol production.**

**(A)** PCA conversion by strain S1 cultivated with 10, 15, 20, and 25 mg/L PCA. **(B)** PCA conversion by strains S2, S3, and S4 cultivated in the presence of 20 mg/L PCA. PCA conversion was determined at 12, 24, 36, and 48 h of cultivation. Data represent the mean ± standard deviation (SD) of three independent biological replicates.
